# Warning and informative messages on e-cigarette package leaflets: A Belgian policy case study for public health policymakers

**DOI:** 10.18332/tpc/217789

**Published:** 2026-07-10

**Authors:** Stijn Everaert, Frieda Matthys, Filip Lardon, Sylvie Gérard, Eline Remue

**Affiliations:** 1 Chemical Environmental Factors Group Superior Health Council Brussels Belgium; 2 Department of Psychiatry Universitair Ziekenhuis Brussel, Vrije Universiteit Brussel Brussels Belgium; 3 Center for Oncological Research University of Antwerp Antwerp Belgium; 4 Mental Health Group Superior Health Council Brussels Belgium; 5 Directorate General Animals Plants and Food, Federal Public Service Health, Food Chain Safety and Environment Brussels Belgium

**Keywords:** leaflet, insert, e-cigarette, prevention, package

## Abstract

The EU Tobacco Products Directive specifies the types of information required on e-cigarette package leaflets, but leaves further details and design to the Member States. In practice, however, both aspects are largely determined by the industry. This policy case study presents a comprehensive leaflet proposal developed by the Belgian Superior Health Council in collaboration with national tobacco control stakeholders. This multidisciplinary engagement enhances both the content quality and its acceptance among health professionals. The proposal defines formatting requirements for maximum legibility and comprehension across the general population, and structures evidence-based warning and informative messages into five thematic chapters. It aims to prevent vaping initiation among non-smokers and specific vulnerable groups by highlighting both short- and long-term health risks, while providing adult smokers with balanced information on the potential role of e-cigarettes in smoking cessation, preferably under professional supervision. The content should be updated periodically to reflect new scientific evidence, and complemented with evaluation metrics to assess real-world impact (leaflet reach, reader comprehension, behavioral influence). Given that leaflets are poorly to moderately noticed and rarely read, integration with pictorial warnings is recommended, following the relatively successful Canadian model for cigarette package inserts.

## Introduction

Initially promoted as a less harmful alternative for smokers, e-cigarettes are increasingly used among never smokers^1^. In Flanders (Belgium), 29% of those aged 12–18 years had tried e-cigarettes by 2023–2024^2^. Nine percent reported vaping at least once a week, which is four times more than 5 years earlier^2^. Since 2018, daily use among those aged 15–24 years has increased tenfold^3^. Driven by curiosity, peer influence and higher risk-taking, young people are specifically targeted by the industry through appealing flavors, attractive product designs, and aggressive marketing strategies^1,4^. Worryingly, vaping may act as a gateway to smoking tobacco, with young e-cigarette users having three times higher chances of smoking initiation^5^. Although e-cigarette aerosols are, puff-for-puff, likely less harmful than tobacco smoke in terms of cytotoxicity and lung inflammation, growing *in vitro*, *in vivo,* and epidemiological evidence points to both acute and chronic health risks^6^.

To address these concerns, public health policymakers are intensifying efforts to curb the ‘vaping epidemic’^4^. All actions within the EU must align with the Tobacco Products Directive (2014/40/EU) (TPD). Belgium has set an ambitious national goal: to achieve a smoke-free generation by 2040, reducing the proportion of daily tobacco users to just 5%. To reach this goal, the *Interfederal Strategy for a Smoke-Free Generation* (2022–2028) was developed. Under this strategy, a political agreement was reached to implement more than 50 concrete actions to foster a healthier, smoke-free society. It stipulates the introduction of standardized package leaflets for both tobacco and herbal smoking products, and e-cigarettes^7,8^. These should provide users with independent information on health risks and information on smoking and vaping cessation. For e-cigarettes, Article 20(4) of the TPD requires EU member states to ensure that unit packets of e-cigarettes and refill containers contain a leaflet with different types of information (e.g. product storage, a reference against use by young people and non-smokers, contra-indications, warnings for specific risk groups, possible adverse effects, addictiveness and toxicity). Currently, implementation is left to the industry, leading to heterogeneous information for consumers. To harmonize this information and guarantee scientific accuracy, Belgian health authorities mandated the Superior Health Council (SHC) to develop standardized, evidence-based warnings and informative messages in consultation with relevant national stakeholders^7^. This policy case study aims to share the Belgian proposal, stimulating international discussion and an exchange of best practices.

## Methodology

This policy case study outlines the efforts by the Belgian SHC to develop standardized, evidence-based content for e-cigarette package leaflets. In 2024, a multidisciplinary working group was established by the SHC, with respect to Article 5.3 of the WHO FCTC. A deontological committee screened all members for potential conflicts of interest. Participants were recruited in national academia, research institutes, tobacco cessation and cancer prevention NGOs, and clinics, covering expertise in analytical chemistry, (respiratory) toxicology, oncology, cancer prevention and screening, general practice, addiction psychology, tobacco control, smoking/vaping cessation, communication, marketing, and consumer behavior.

Warning and informative messages were developed through a consensus process over multiple discussion rounds (October 2024 – July 2025). The discussions were based on previous positions of the Council^7,9^, updated by a targeted narrative literature review of new data in PubMed^7^, and national stakeholder experience.

## Proposal

### Aim

The content of the leaflet must be clear and concise, addressing two aims: Preventing vaping initiation among non-smokers and vulnerable groups by highlighting risks, and secondarily providing adult smokers with independent information about the potential role of e-cigarettes as a cessation aid for conventional cigarettes.

### Formatting requirements

For maximum legibility and to distinguish the warning and leaflet messages from manufacturers’ information, a clear typeface, larger font, distinct color, and adequate line spacing are recommended. The standardized, mandatory messages should be placed prominently at the beginning of the leaflet, preceding all industry information. Plain language is needed to guarantee equitable access for all users, regardless of educational and socio-economic backgrounds. General information should appear first, followed by tailored information for specific (user) groups. The content should be periodically revised to integrate new scientific evidence and updated regulatory data. The messages may be strengthened by incorporating pictorial warnings, aligning with evidence from the Canadian cigarette package inserts^10-12^.

### Content

Different messages are proposed (Table 1), subdivided into five thematic categories.

**Table 1:** Proposed warning and informative messages for e-cigarette package leaflets, developed by the Belgian Superior Health Council (2025) and translated from Dutch and French. Messages marked with * apply only to nicotine-containing e-liquids

**1. General message**	Healthy living = no smoking, no vaping. E-cigarettes are strongly discouraged for non-smokers, especially youths and young adults (<25 years) and pregnant women. E-cigarettes can be used as a possible aid for adult smokers to quit smoking, preferably under the supervision of a health professional.
**2. Health risks**	Various chemical compounds of this product are harmful to health. This product contains nicotine. Its use is not recommended.* Some possible health effects of e-cigarettes In the short-term, there is an increased risk of: irritation of the throat, eyes, and respiratory tract. respiratory complaints such as coughing and pneumonia-like symptoms. asthma attacks and exacerbations. certain changes in blood vessel function. In the long-term, there is an increased risk of: damage to the respiratory tract. serious lung diseases such as COPD. DNA damage, which can contribute to the development of certain cancers. adverse effects on fetal development and at birth (e.g. low birth weight, premature birth). effects that are currently unknown. Additional risks specifically associated with nicotine-containing e-cigarettes*: highly addictive. increased heart rate and blood pressure. cardiovascular disease (e.g. heart attack). possible psychological effects such as irritability, stress, anxiety, depression, loss of concentration, and sleep disorders (however, individual perceptions may vary).
**3. Specific risk groups (pregnant women, youths, young adults<25 years, and people with underlying conditions)**	The Belgian law prohibits the sale and offering of e-cigarettes to persons under 18 years of age. E-cigarette use is strongly discouraged for youths and young adults (<25 years), pregnant women, and breastfeeding women. Exposure of the fetus to chemicals is harmful and can lead to disease later in life. Exposure of youths and young adults (<25 years) to chemicals can be harmful to brain development. Keep out of reach of children. Fatal to children if swallowed and/or in contact with skin*. The use of e-cigarettes is strongly discouraged for people with: lung, cardiovascular, kidney, or liver problems, diabetes, psychiatric and other chronic conditions. people with inflammation of the mouth, throat, or esophagus. hypersensitivity or allergy to one or more ingredients.
**4. The e-cigarette with nicotine as a potential smoking cessation aid**	According to current knowledge, e-cigarettes are less harmful than smoking tobacco when used temporarily (<1 year). E-cigarettes do not produce tar and much less carbon monoxide, two of the most harmful components of tobacco smoke. However, e-cigarettes are not harmless. The consequences of long-term use (>1 year) are insufficiently known. Only use e-cigarettes to quit smoking. How to quit smoking using e-cigarettes? Preferably seek guidance from your doctor, pharmacist, tobacco cessation specialist (*tabakologen.be*) or Tabakstop (*tabakstop.be* or quitline 0800 111 00). It is preferable to start with nicotine levels in the e-liquid that match your (previous) tobacco use. Gradually reduce the nicotine level until you can also stop using e-cigarettes. Alternating between vaping and smoking offers no health benefits, so quit smoking completely. You may experience withdrawal symptoms when you stop vaping nicotine. This should not cause you to start using tobacco cigarettes again. Follow the standard advice that is also given for smoking cessation.
**5. Product risks**	Do not buy e-cigarettes or e-liquids online. It is illegal and unreliable, as there are no safety guarantees. Never use oil-based e-liquids, they are dangerous. Avoid overheating (dry puffs). A bitter taste indicates the presence of carcinogenic substances and additional health risks.

#### Category 1: General message

Three statements represent the overall position of the SHC towards the e-cigarette^7,9,13^. While vaping is regarded as a threat for non-smokers and vulnerable groups^1,6,14^, the potential of the e-cigarette as a smoking cessation aid is acknowledged^15,16^.

#### Category 2: Health risks

Vapers are exposed to a complex aerosol of propylene glycol and vegetable glycerin, carrying nicotine (salts), numerous flavorings, thermal degradation and interaction products (including toxic aldehydes, furans, free radicals, etc.), and even metals released from the device^15,17^. While most flavorings are considered safe for ingestion as food additives, their inhalation toxicity is insufficiently known^13,17^. Several flavorings induce oxidative stress and exhibit cytotoxic, genotoxic, and pro-inflammatory effects in, amongst others, human respiratory epithelial cells^6,13,15,17^. Robust evidence exists for multiple adverse health outcomes^1,6^.

The messages point out the risk of chemical exposure, while differentiating between short- and long-term effects of vaping, and the specific effects of nicotine. In addition to respiratory effects, cardiovascular, carcinogenic, psychological, and adverse birth outcomes are addressed^1,6,7,9,13-15,17-23^. For example, the risk of severe lung diseases like COPD is significantly increased^18,19^, while elevated biomarkers for genotoxicity and oxidative stress are linked to cancer risks^14^.

The main challenge is presenting health effects in plain language for non-medical readers, without losing scientific accuracy. For example, the general phrasing ‘*certain changes in blood vessel function*’ (after short-term exposure) refers to complex changes in endothelial function after vaping nicotine-free e-cigarettes, observed via MRI^22^.

#### Category 3: Specific risk groups (pregnant women, youths, young adults <25 years, and people with underlying conditions)

Prenatal and early postnatal life is highly vulnerable to chemical exposure, which may not only affect early development, but also increase the risk of various adult diseases decades later^24^. In mice, maternal e-cigarette exposure alters DNA methylation and lung cytokine expression in the offspring^25^. Prenatal exposure to vanilla-flavored e-cigarette aerosols results in sex-specific alterations of the lung transcriptome at birth, increasing the asthma susceptibility of the mice offspring^26^. In humans, prenatal vaping is associated with 53% higher odds of adverse neonatal (including low birth weight, preterm birth, and small for gestational age) and maternal outcomes (including decreased breastfeeding)^23^. Given the toxicological complexity of e-cigarette aerosols, the warning messages intend to prevent early exposure. As the prefrontal cortex continues to develop and mature around the age of 25 years, this age is set as the upper limit of vulnerability^27^. As some e-cigarette toxicants may impact breast milk^28^, a warning is included to discourage vaping during lactation, based on the precautionary principle.

In addition, the leaflet also advises against vaping for people with chronic conditions (lung and cardiovascular diseases, kidney or liver problems, diabetes, etc.), inflammation of the mouth, throat, esophagus, or in case of hypersensitivity or allergy to one or more ingredients.

#### Category 4: The e-cigarette with nicotine as a potential smoking cessation aid

According to the Cochrane living review, high-certainty evidence exists that e-cigarettes with nicotine increase smoking quit rates (8–11 of 100 people) compared to nicotine replacement therapy (NRT, 6 of 100 people)^16^. Based on these data and the popularity of the e-cigarette as a smoking cessation aid in Belgium (23.7%), before NRT (12.2%)^29^, the Belgian SHC still acknowledges the potential of e-cigarettes as a smoking cessation aid, preferably under the supervision of a health professional. However, this position remains debated: while embraced by the British Royal College of Physicians^15^, it is rejected by the WHO^4^ and the European Respiratory Society^30^.

The proposed messages stipulate that e-cigarettes should only be used to quit smoking. Due to the absence of tobacco combustion, vaping is likely less harmful than smoking (on a puff-for-puff basis), especially when used temporarily, while long-term effects are insufficiently known^6^. Practical tips for smoking cessation are provided, with first and foremost a referral to professional cessation guidance. Dual use is explicitly discouraged, as it increases risks for acute respiratory infections^6^, and significantly increased pooled ORs for several other outcomes (cardiovascular disease, stroke, metabolic dysfunction, asthma, COPD, oral disease)^1,6,31^. It is reiterated that one should also quit vaping after successful smoking cessation. Nicotine withdrawal symptoms should not prompt relapse to combustible cigarettes.

#### Category 5: Product risks

Finally, the messages mention that no safety guarantees exist for illegally purchased vapes. Some other product risks are highlighted, including dangers of oil-based e-liquids (e.g. lipoid pneumonia^32^), and toxic aldehydes generated by thermal decomposition of the e-liquid ingredients during dry puffs. Dry puffs occur when the power settings are too high, or in case of insufficient e-liquid saturation^33^.

### Strengths and limitations

The main strength of this leaflet proposal is the support of professionals in the field. It is an adaptive tool to reach smokers and vapers with reliable information. Its limitations are the pending implementation by lawmakers, the time it will take to adapt messages, and the need for evaluation metrics to measure real-world impact (leaflet reach, reader comprehension, influence on vaping behavior). In general, package leaflets are seldom noticed. In a study on e-cigarette use by current e-cigarette smokers/quitters in six EU countries in 2018, 11.6% noticed the leaflets, while only 3.97% read the information^34^. Similarly low numbers were observed in England (19.1% noticed leaflets in 2018)^35^ and the Netherlands (33.4% noticed leaflets in 2017)^36^. While the Dutch study noticed a slight increase in risk perception after the implementation of the TPD in 2016^36^, concerns did not change significantly in England^35^. Overall, the impact remained low^36^.

### Implications

It is clear that in order to increase leaflet effectiveness, the messages on the leaflets must be combined with other actions: price setting, flavor restrictions, supply regulation, marketing, and a broader societal prevention strategy. In Canada, higher efficacy was reported when cigarette package leaflets (inserts) were combined with prominent pictorial warnings^10-12^. In a 2 week randomized trial, participants whose packs included leaflets were more likely to report foregoing or stubbing out cigarettes (OR=2.39; 95% CI: 1.36–4.20) than those without leaflets^11^. The frequency of leaflet reading was associated with self-efficacy to quit smoking, more attempts to quit, and sustained cessation at follow-up^10^. It is likely that these findings might also apply to e-cigarette leaflets.

## Conclusion

While the TPD specifies the information types required on e-cigarette package leaflets, further details and design are left to the EU Member States. In practice, however, the industry is designing the leaflet. This policy case study presents a comprehensive leaflet proposal for lawmakers, developed in collaboration with Belgian tobacco control stakeholders. It presents standardized, clear, reliable information on health risks for non-smokers and specific vulnerable groups, alongside guidance on product use for smoking cessation. The content should be evaluated over time, based on new insights into scientific accuracy and effectiveness. To enhance the latter, combination with pictorial warnings is recommended, as evidenced by the Canadian experience with cigarette package leaflets.
